# Epidemiological Studies to Support the Development of Next Generation Influenza Vaccines

**DOI:** 10.3390/vaccines6020017

**Published:** 2018-03-26

**Authors:** Joshua G. Petrie, Aubree Gordon

**Affiliations:** Department of Epidemiology, University of Michigan School of Public Health, Ann Arbor, MI 48109, USA; jpetrie@umich.edu

**Keywords:** influenza, influenza vaccines, epidemiology, correlates of protection, hemagglutinin, neuraminidase

## Abstract

The National Institute of Allergy and Infectious Diseases recently published a strategic plan for the development of a universal influenza vaccine. This plan focuses on improving understanding of influenza infection, the development of influenza immunity, and rational design of new vaccines. Epidemiological studies such as prospective, longitudinal cohort studies are essential to the completion of these objectives. In this review, we discuss the contributions of epidemiological studies to our current knowledge of vaccines and correlates of immunity, and how they can contribute to the development and evaluation of the next generation of influenza vaccines. These studies have been critical in monitoring the effectiveness of current influenza vaccines, identifying issues such as low vaccine effectiveness, reduced effectiveness among those who receive repeated vaccination, and issues related to egg adaptation during the manufacturing process. Epidemiological studies have also identified population-level correlates of protection that can inform the design and development of next generation influenza vaccines. Going forward, there is an enduring need for epidemiological studies to continue advancing knowledge of correlates of protection and the development of immunity, to evaluate and monitor the effectiveness of next generation influenza vaccines, and to inform recommendations for their use.

## 1. Introduction

With recent publication of their strategic plan for the development of a universal influenza vaccine, the National Institute of Allergy and Infectious Diseases (NIAID) has clarified the criteria that a universal vaccine should meet and has prioritized research to fill the gaps in knowledge required to successfully develop and implement such a vaccine [1]. The minimal desired characteristics of a universal vaccine are that it be ≥75% effective in preventing symptomatic influenza infection, that it provides broad protection against hemagglutinin (HA) group I and II influenza A viruses, and that it provides protection that persists for at least one year. These desired characteristics provide clear goals for development of new vaccines, but also highlight current gaps in knowledge and areas where our understanding of current influenza vaccines will be important. To achieve the goal of universal vaccine development, NIAID has recognized the need for research to better understand (1) influenza transmission, natural history, and pathogenesis; (2) development of influenza immunity and correlates of protection; and (3) rational design of universal influenza vaccines. Epidemiological studies have played and will continue to play an important role in addressing each of these three related research areas. The continued need for epidemiological studies is highlighted by the call for longitudinal cohort studies in NIAID’s strategic plan.

Here, we discuss the contributions of epidemiological studies to our current knowledge of vaccines and correlates of immunity, and how they can contribute to the development and evaluation of the next generation of influenza vaccines. These studies have been critical in monitoring and identifying shortcomings in the effectiveness of current influenza vaccines. Epidemiological studies have also identified population-level correlates of protection that can inform the design and development of next generation influenza vaccines. Going forward, there is a continued need for epidemiological studies to understand influenza transmission and natural history of infection, to characterize the development of immunity following natural infection and vaccination, to evaluate and monitor the effectiveness of next generation influenza vaccines, and to inform recommendations for their use.

## 2. Effectiveness of Current Influenza Vaccines

It has become apparent over recent years that the effectiveness of current influenza vaccines is insufficient. In the context of tens of thousands of deaths and hundreds of thousands of hospitalizations in United States each year [2], vaccine effectiveness against influenza A(H3N2) has been estimated to be less than 50% in each of the last six influenza seasons [3,4,5,6,7,8]. Although the number of influenza illnesses and hospitalizations that can be averted is substantial [9], even with a vaccine that has limited effectiveness, it is clear that improved vaccines are needed. As progress is made toward a more broadly protective influenza vaccine, or a universal vaccine, it is critical that the problems associated with the current vaccine are understood. Foremost among the problems with current influenza vaccines are low vaccine effectiveness, even lower vaccine effectiveness among those who receive repeated vaccination, and egg-adaptation mutations occurring during the manufacturing process may change the antigenicity of the vaccine strain viruses relative to that of the circulating viruses. These issues may all be interrelated through the effects that pre-existing antibodies in the population have on response to subsequent vaccination. Immune histories might also be expected to affect the response to and effectiveness of next generation vaccines if not carefully considered during design, evaluation, and implementation.

Over the past decade, many countries have established epidemiological programs to monitor annual influenza vaccine effectiveness. Meta-analysis of these recent studies show variability by influenza type and subtype with the highest average annual vaccine effectiveness against influenza A(H1N1)pdm09 (61%) and lowest (33%) against influenza A(H3N2) [10]. There is also significant variability in effectiveness from year to year, including the 2014–2015 season in which the vaccine was completely ineffective in preventing antigenically drifted influenza A(H3N2) viruses [5]. While antigenic mismatch between vaccine strains and circulating viruses can be a major determinant of low vaccine effectiveness, it is clear that this is not the only factor. For example, vaccine effectiveness against influenza A(H3N2) during the 2016–2017 season was only 43% in the United States even though the vaccine was considered to be well matched to circulating viruses [7].

A variety of potential causes, in addition to antigenic match, have been proposed to explain low vaccine effectiveness. As described below, a common theme is that all are impacted by the epidemiology of population immunity developed through past vaccination and infection. Integration of immunological studies with ongoing epidemiological studies of influenza vaccine effectiveness is essential to understanding these issues and for the development of next generation vaccines. This could be accomplished through collection of acute and convalescent serum specimens from individuals enrolled in vaccine effectiveness studies or through population-based studies of influenza vaccine response carried out in the same communities.

## 3. Effectiveness of Repeated Influenza Vaccination

In some seasons, reduced vaccine effectiveness has been associated with repeated vaccination. Specifically, when this effect is observed, those vaccinated only in the current season have higher vaccine effectiveness relative to those who were vaccinated in both the current and prior seasons. It is also important to note that those unvaccinated in both current and prior seasons are typically at highest risk of infection, and that residual protection has frequently been observed among those vaccinated only in the prior season [11]. These are not new observations, but rather a long-standing question [12,13] reignited by findings over the past decade that have been facilitated by increased monitoring of the performance of influenza vaccines [11,14]. It is of note that reduced vaccine effectiveness following repeated vaccination has not been observed in all influenza seasons and has been most frequently associated with influenza A(H3N2). It has been recognized that the variability in these observations is not evidence for lack of an effect, but rather point to a complex underlying immune mechanism [11].

Antibodies to the influenza HA globular head are recognized as the primary correlate of protection against influenza infection, and current influenza vaccines are designed to elicit in vaccinated individuals an antibody response that is targeted to the HA head. Reduced hemagglutination-inhibition (HAI) antibody response following vaccination has consistently been observed among those who have been previously vaccinated compared to those who have not [15]. However, absolute post-vaccination titers may or may not ultimately be lower among those repeatedly vaccinated. Recent household cohort studies have observed lower post-vaccination HAI titers among those with repeat vaccination compared to those vaccinated only in the current season in years when vaccine effectiveness is reduced with repeat vaccination [16], but no differences in titer have been observed in seasons when a repeat vaccination effect is not evident in vaccine effectiveness estimates [17,18]. As with the overall patterns of vaccine effectiveness following repeat vaccination, the variability in the response to vaccination and absolute post-vaccination titer points to a complex underlying immune mechanism. In a seminal paper, Smith et al. proposed the antigenic distance hypothesis to reconcile the variability in the observed effectiveness of repeated vaccination [19]. This hypothesis proposes that the antigenic relatedness of the vaccine strain given in one year (v1) to the vaccine strain given in the following year (v2) and to the epidemic strain that circulates in the second year (e) determine whether or not reduced protection against e will be observed among those consecutively vaccinated. If v1 and v2 and e are all antigenically similar, reduced effectiveness of v2 would not be expected. However, if v1 and v2 are antigenically similar, but e is sufficiently distinct from v1, reduced protection against infection would be expected among those vaccinated in consecutive years.

Observed patterns of vaccine effectiveness following repeat vaccination have been generally consistent with this hypothesis, which nevertheless does not completely explain the observed effects. One difficulty in assessing this hypothesis is limitations in how antigenic relatedness of influenza viruses is determined. The most common method to determine antigenic relatedness is by measuring the ability of post-infection ferret serum to neutralize or inhibit hemagglutination of panels of past and contemporary circulating influenza viruses. The panels of ferret serum used are harvested from ferrets that have been infected by a single virus, and the population of antibodies present is likely much different from that of a human with an extensive history of exposure to various influenza antigens through infection or vaccination. There is a critical need for expanded integration of surveillance and epidemiological studies of the specificity of the human antibodies to annual circulating influenza viruses to inform development of next generation influenza vaccines and improve strain selection for current influenza vaccines. It is becoming clear that distinct immune histories can cause these specificities to vary across individuals.

## 4. Epitope Focusing

It has long been established that the existing immunity developed through past influenza exposure can shape the immune response to subsequent vaccination or infection [20,21]. However, understanding of the granularity by which this occurs is beginning to develop. Recent studies have demonstrated that the antibody response to influenza vaccination is not homogenous, but rather represents a population of antibodies with varying specificities to different epitopes of the influenza HA [22]. It has been posited that repeated exposure to antigenically related viruses selects for B cell populations (which give rise to these antibodies) that are narrowly focused in their recognition of epitopes that are conserved between different, but related viruses [23]. This is not a problem for protection per se, but rather reduces the breadth of immunity such that circulating viruses can evade the antibody response by mutations that change the phenotype of a single or few epitopes.

Such a scenario was observed during the 2013–2014 season in which influenza A(H1N1)pdm09 viruses predominated. Nearly all viruses that circulated in this season possessed a K166Q mutation in the HA which was not present in the A/California/7/2009 strain included in the 2013–2014 vaccine and all prior influenza vaccines since the 2009 pandemic [24]. Despite this mutation, antigenic characterization using ferret antisera suggested that 2013–2014 circulating A(H1N1)pdm09 viruses remained similar to the A/California vaccine strain [25]. However, some individuals produced antibody which recognized the A/California vaccine strain but not viruses differing only by the K166Q mutation suggesting narrow focusing of available antibodies to this epitope that differed between vaccine and circulating strain viruses [26]. Middle-aged individuals were most likely to have this antibody specificity, and this was consistent with reports of increased incidence of infections within these age groups [27]. Further research confirmed that antibodies against viruses with the K166Q mutation better correlated with protection from laboratory-confirmed infection than antibodies against the vaccine strain virus [28].

There has also been research demonstrating focusing of the antibody response to changes in influenza HA epitopes that arise as a result of egg-adaptation during the vaccine manufacturing process. Because most influenza vaccines are produced in hens’ eggs, the vaccine seed viruses are adapted for efficient growth in eggs. This results in phenotypic changes to the virus, but does not always obviously contribute to reduced effectiveness of the vaccine. However, poor performance of the vaccine against influenza A(H3N2) in the 2016–2017 and 2017–2018 seasons has been linked to antibody focusing toward epitopes associated with egg-adaption [29]. Influenza A(H3N2) viruses that have circulated since the 2014–2015 season acquired a new glycosylation site in the HA antigenic site B relative to A(H3N2) viruses that had circulated in the recent past [30]. The egg-adapted A(H3N2) virus included in the 2016–2017 and 2017–2018 influenza vaccines acquired a reversion mutation, resulting in loss of this new glycosylation site [31]. Age-related differences have been demonstrated in the ability of the vaccine to induce an antibody response that can effectively bind circulating viruses [32]. This suggests that, in some individuals, past exposure shapes the antibody response such that it is narrowly focused to HA epitopes that are available in the vaccine, but shielded in circulating viruses. It is possible that this has contributed to the poor performance of the influenza vaccine against influenza A(H3N2) in these two seasons [7,8].

These recent examples illustrate the need for longitudinal studies to increase understanding of how influenza immunity develops after infection or vaccination and to identify additional, more broadly protective correlates of immunity. These studies will need to be targeted by age group. It is essential to understand how immunity develops in infants and young children following their first exposures in life, and the differences in immunity produced following infection and vaccination. It is also essential to understand the dynamics and specificity of the immune response in both younger adults and the elderly who have varying immune histories based on exposure throughout their lives.

## 5. Implications for Next Generation Vaccines

While influenza vaccine effectiveness has been suboptimal across multiple seasons, particularly against influenza A(H3N2), the reasons for low effectiveness have varied. In addition to the antigenic match between vaccine strain and circulating viruses, issues related to repeat vaccination, egg adaptation, and antibody specificities shaped by past infection and vaccination have all played a role. The magnitude of the effect of each of these factors is ultimately determined by the epidemiology of infection, vaccination, and pre-existing antibody in specific populations.

These effects may continue to be relevant for next generation influenza vaccines, particularly those that do not establish lifelong immunity given that current goals continue to aim for duration of protection of at least one year. Therefore, a thorough understanding of the development of influenza immunity and monitoring of the dynamic response to next generation vaccines over time is required. Some next generation vaccines may continue to target the globular head of the influenza virus HA by focusing on eliciting broader or longer lasting immunity through use of adjuvants, or improving antigenic match to circulating viruses, and thus, effectiveness, through production methods that do not involve eggs. However, development of next generation vaccines may also focus on the development of antibodies directed toward other influenza virus antigenic sites. Because these sites may be more conserved, there is a potential that these vaccinations could provide universal protection, at least against influenza A viruses.

## 6. Anti-Hemagglutinin Stalk Antibodies

In contrast to the globular head domain of HA, which is the target of currently licensed inactivated season influenza vaccines, the stalk domain is more conserved. A number of broadly neutralizing anti-HA stalk antibodies have been identified following natural influenza A infection that target either group 1 (H1, H2, H5, H6, H8, H9, H11, H13, H16, H17, H18) or group 2 HAs (H3, H4, H7, H10, H14, H15) [33,34,35,36]. The prevalence of broadly neutralizing anti-HA stalk antibodies has been found to be high in multiple adult populations [37,38]. However, the seasonal influenza vaccine typically does not elicit a strong anti-HA stalk response, except when the vaccine contains a HA antigen that is significantly different from prior seasonal strains the individual has encountered [34,35,39,40,41,42].

It has been hypothesized that broadly neutralizing anti-HA stalk antibodies are in part responsible for seasonal influenza virus replacement following a pandemic when viruses are from the same HA group [43]. Consistent with this hypothesis, anti-HA stalk antibodies were boosted in individuals infected with influenza A(H1N1)pdm09 during the 2009 pandemic, and the seasonal influenza A(H1N1) subtype that circulated prior to the pandemic has not been detected since [44]. The conservation of the stalk domain across influenza A groups and the identification of naturally occurring broadly neutralizing antibodies has led to significant interest in the stalk as a potential target of a universal influenza vaccine. Animal models clearly support that stalk antibodies can provide protection from influenza, but data on how anti-HA stalk antibodies correlate with protection in humans is limited.

A healthy volunteer study demonstrated that individual anti-HA stalk response occurs in a majority of adults after infection with influenza A(H1N1)pdm09, but varied significantly following infection. Anti-HA stalk antibody level was correlated with a reduction in detectable viral shedding, but was not correlated with a reduction in clinical symptoms [38]. Further studies are needed to evaluate anti-stalk antibodies as a correlate of protection and to investigate their effect on influenza transmission. Such studies will need to include longitudinal cohorts that span all ages. Cohorts with enhanced transmission surveillance such as household cohorts that intensively monitor for transmission or other group settings will likely be especially informative. Transmission studies in households, schools, or other group settings should also be informative.

## 7. Anti-Neuraminidase Antibodies

Although current seasonal influenza vaccines focus on raising an immune response against the HA head, there is increasing evidence that antibodies directed toward influenza neuraminidase (NA) also contribute to protection. Amounts of NA antigen in seasonal influenza vaccines vary, but is typically quite low and is not regulated. Proposals to standardize the amount of NA in the seasonal influenza vaccine formulations have been discussed, but have never been adopted due to both regulatory and stability concerns as well as uncertainty over whether an enzymatically active NA is necessary. For many years, the salient issue was the lack of a standardized, high-throughput assay to assess anti-NA antibody as a correlate of protection; however, with the advent of enzyme-linked lectin assay (ELLA), that issue has been addressed [45]. Multiple vaccine studies have demonstrated that it is possible to generate high NA inhibition (NI) seroconversion rates from vaccination with vaccines containing a sufficient quantity of NA antigen [46,47,48].

Several epidemiological studies have found that anti-NA antibodies provide protection against natural influenza infection or illness. In the Tecumseh study of respiratory illness, during the 1968 H3N2 pandemic, individuals with pre-existing anti-NA antibody titers from prior H2N2 infection had lower H3N2 serologically confirmed infection rates than individuals without detectable titers. Further, anti-NA antibodies may have reduced the severity of clinical presentation among those who were infected with influenza [49]. More recently, a study found that NI antibodies from prior natural infection were an independent correlate of protection from influenza infection and illness [50]. Another study in Michigan found that while only approximately one-third of inactivated influenza vaccine recipients experienced a ≥4-fold rise in NI antibody following vaccination, NI antibodies appear to have an independent role in protection against PCR-confirmed symptomatic H3N2 infection in both vaccinated and unvaccinated individuals [51]. In a recent human challenge study, NI titers were negatively correlated with duration of viral shedding, duration and number of symptoms, and severity in influenza A(H1N1)pdm09 infections [52]. Indeed, the authors found that protection was associated more strongly with NI titer than with HAI titer; however, it is important to note that influenza illness in this study was significantly less severe than typically observed in community settings [52,53].

While the above studies provide evidence that anti-NA antibodies correlate with both protection from clinical illness and disease severity, and that the protection likely extends to heterologous viruses, important questions remain that need to be addressed through epidemiological studies. All of these studies provide evidence that anti-NA antibodies are a correlate of protection in healthy adults aged 18 to 50 years; however, none include children or the elderly, two extremely important groups for vaccination. Longitudinal cohorts or community transmission studies that span the entire lifespan are needed to evaluate NI titers as a correlate of protection in children and the elderly. Longitudinal cohorts will also play an important role in characterizing the development of immunity to NA and investigating antigenic drift of NA.

Whether NA will be a target of next-generation influenza vaccines or a universal influenza vaccine remains an open question, however, epidemiological evidence suggests that the inclusion of NA in seasonal influenza vaccines could be beneficial.

## 8. Immune History and Next Generation Vaccines

As discussed above, immune history affects response to seasonal influenza vaccination and clearly affects response to natural infection. The concept of original antigenic sin was introduced around 70 years ago when researchers noted that an individual’s antibody response to influenza virus is dominated by strains encountered in childhood [20,54,55]. More recently, researchers have coined the term antigenic seniority to describe the stronger antibody response to viruses encountered in childhood than to contemporary viruses that does not necessarily come at the expense of the response to the contemporary viruses [56,57]. One possible mechanism for this is that antibody titers may increase at a similar rate longitudinally, therefore viruses encountered earlier in life accumulate to the highest levels [58]. This phenomenon has been observed in multiple studies [54,56,57,58], and may not be exclusive to anti-HA antibodies as it has been noted for anti-NA antibodies as well [59].

A recent modeling study indicated that HA imprinting may have strong effects related to susceptibility to severe illness across HA groups. Specifically, the authors of that study found that individuals first exposed to H1N1 or H2N2, group 1 viruses, were spared from severe infection and death by avian H5N1, another group 1 virus, and likewise, individuals infected with H3N2, a group 2 HA virus, early in life were protected from severe or fatal avian H7N9 infections, another group 2 virus [60]. The same effect was not noted for NA imprinting. These data strongly support that viral exposures early in life have important and life-long effects on influenza immunity, protecting against future novel viruses. In addition, the effect of HA imprinting will need to be considered carefully in the design of a universal vaccine given that HA imprinting will vary in the population.

Although original antigenic sin was first described seven decades ago, there is still a very incomplete understanding of how first influenza exposures shape antibody response throughout the lifetime. Longitudinal cohort studies provide an opportunity to determine how these first exposures affect subsequent response to infection or vaccination. Because influenza A(H3N2) and A(H1N1)pdm09 are currently co-circulating there are age cohorts whose first exposure may be either through infection with one subtype or the other, or through vaccination including both subtypes. Thus, the potential confounding effects of age can be addressed. Although evidence currently supports that it is the first exposure to influenza A that determines HA imprinting, existing studies have not had the ability to differentiate between early life exposure and first exposure. Longitudinal cohort studies in infants and children will provide a unique opportunity to characterize the immune response to first infection including HA imprinting and the effect of these first exposures on subsequent clinical risk as well as the immune response to repeat infections or vaccination. To do so, it will be important for immunologists to work with epidemiologists to inform appropriate specimen collection and analytic strategies. Cohorts that span all age ranges, particularly family cohorts, can be useful for studying susceptibility and response to similar viruses in individuals with a broad range of immune histories.

## 9. Conclusions

As the next generation of influenza vaccines are developed, there is much to learn from past and ongoing epidemiological studies of influenza vaccine effectiveness and humoral correlates of protection. Immune histories shaped by past infection and vaccination influence individuals’ response to subsequent vaccination. This has affected the performance of current influenza vaccines, and will likely affect the next generation of influenza vaccines, particularly in the likely case that more than a single dose is needed in a lifetime. Because immune histories vary within the population, all new vaccines should be evaluated across broad age ranges and among individuals with a range of cumulative prior vaccination and infection. Once the next generation of influenza vaccines are licensed, there will be a need for continued monitoring of their effectiveness in the context of evolution of circulating influenza viruses and changes in population levels of immunity. In these future evaluations, as well as in ongoing evaluations of current influenza vaccines, it is critical to integrate immunologic assessments with epidemiological studies.

These integrated epidemiological and immunologic investigations will be essential to meeting research priorities to support the development of a universal influenza vaccine, particularly the first two focus areas: (1) improved understanding of influenza transmission, natural history, and pathogenesis; and (2) characterization of development of influenza immunity and correlates of protection [1]. Longitudinal cohorts, along with transmission studies in households or other group settings, provide an opportunity to better understand influenza transmission, natural history, and pathogenesis. In addition, they provide a unique opportunity to examine the varying immune response of individuals from different age cohorts, and thus different immune histories, to the same virus strain. Development of immunity can be characterized both in terms of response to a single infection or vaccine as well as in the context of repeated vaccination and infection throughout the life course. Population-based or risk group specific studies of immunologic response to annual vaccination can be used to address the first scenario, while longitudinal cohorts carried out across broad ages will be important in characterizing longer term development of immunity. Longitudinal cohorts, transmission studies, and evaluations of acute and convalescent serum from VE studies can also contribute to better characterization of immunologic correlates of protection against influenza infection. Expanded support of these types of epidemiological studies is essential to understanding the shortcomings of current influenza vaccines and supporting the development and use of the next generation of vaccines.
